# The Honeycomb illusion: Uniform textures not perceived as such

**DOI:** 10.1177/2041669516660727

**Published:** 2016-07-25

**Authors:** Marco Bertamini, Michael H. Herzog, Nicola Bruno

**Affiliations:** University of Liverpool, UK; Brain Mind Institute, Ecole Polytechnique Federale de Lausanne (EPFL), Lausanne, Switzerland; Università di Parma, Italy

**Keywords:** Texture, crowding, peripheral vision, suppression, Honeycomb illusion, Extinction illusion

## Abstract

We present a series of patterns, in which texture is perceived differently at fixation in comparison to the periphery, such that a physically uniform stimulus yields a nonuniform percept. We call this the *Honeycomb illusion*, and we discuss it in relation to the similar Extinction illusion (Ninio & Stevens, 2000). The effect remains strong despite multiple fixations, dynamic changes, and manipulations of the size of texture elements. We discuss the phenomenon in relation to how vision achieves a detailed and stable representation of the environment despite changes in retinal spatial resolution and dramatic changes across saccades. The Honeycomb illusion complements previous related observations in suggesting that this representation is not necessarily based on multiple fixations (i.e., memory) or on extrapolation from information available to central vision.

We report the *Honeycomb illusion*: a phenomenon about visual awareness of how a surface varies across the visual field. The illusion is easy to observe and is illustrated in Figure 1. However, note that on a printed page, these images are likely to appear too small. To best experience the illusion, each image needs to extend to a large proportion of the visual field. For example, it works well when printed on an A4 page and looked at from a distance of about 65 cm (the width of the page covering ∼18° of visual angle). On a computer monitor, the same can be achieved by enlarging the figure to fill most of the screen. An online version can be seen on the Best Illusion of the Year contest website: www.illusionoftheyear.com/2015/the-honeycomb-illusion/, and higher resolution images are available on the Open Science Framework project page: https://osf.io/kabyz/

**Figure 1.**
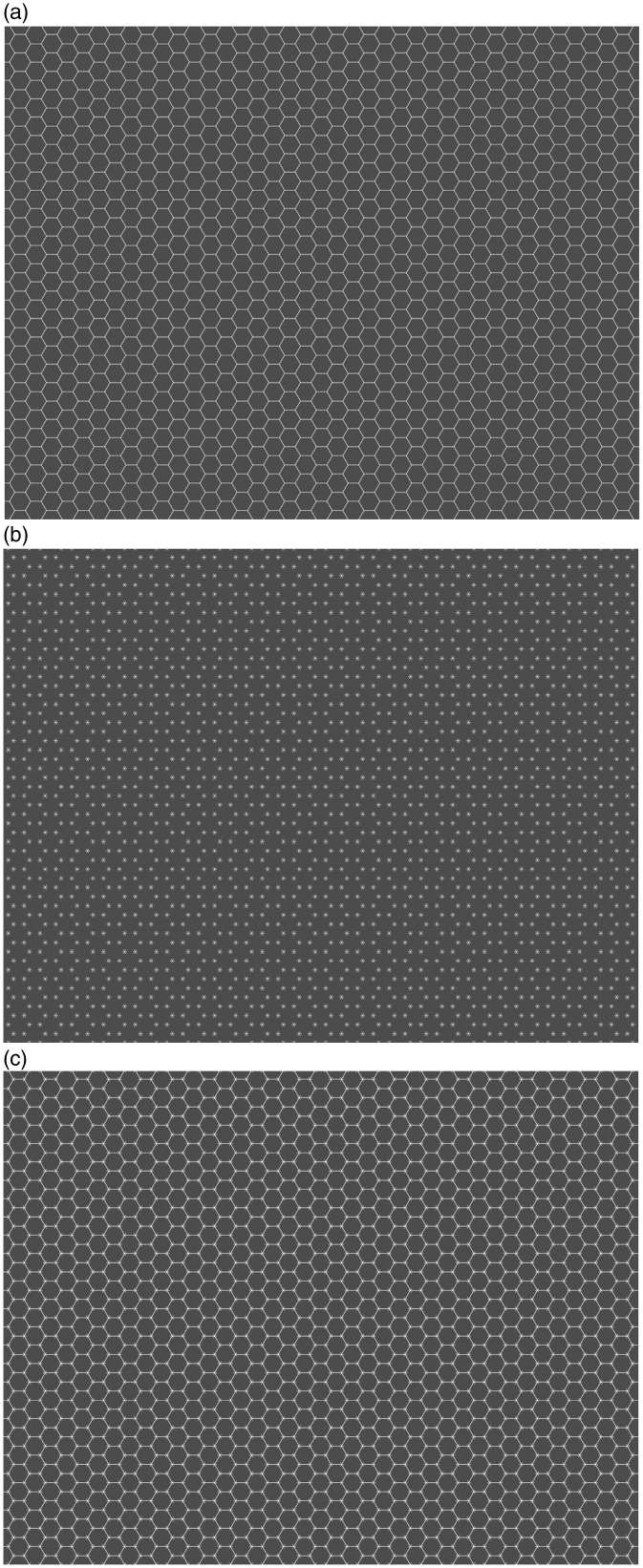
Images need to be seen enlarged (at least 18° of visual angle), for more online images go to: https://osf.io/kabyz/ (a) This is a texture with only hexagons. The texture is (and looks) uniform. (b) This is a texture with lines (barbs). The texture is (and looks) uniform. (c) This is a combination of the previous two textures. The texture is uniform but does not look uniform, for the barbs are perceived only around fixation.

The first two images in Figure 1 show two components that are then combined in the third image. These are hexagons (A) and six small lines (*barbs*) at the vertices of invisible hexagons (B). One can observe that the texture is uniform and is perceived as uniform in both A and B. However, in C, the texture is uniform, but it is not perceived as such. The barbs are visible at fixation but are gone everywhere else. This should be easy to observe. For instance, try holding a steady gaze on the center and then count how many hexagons have lines at the corners. You will notice that only a few hexagons around fixation appear to have lines, while in the periphery the pattern is perceived as made up of regular hexagons (without barbs).

There are several reports in the literature about illusory percepts that become visible away from fixation: the Hermann Grid (Hermann 1870), the Bergen Grid (Bergen, 1985), the Scintillating Hermann Grid (Schrauf, Lingelbach, & Wist, 1997), and the Gingham illusion (Tyler, 2000). In these cases, an illusory percept is generated in the periphery (patches in the Hermann Grid case, scintillating corners of squares in the Gingham illusion). We will return to these phenomena in the Discussion section. It is clear that “illusions, even when they are given different names, often seem to have strong family ties and form a continuum” (Ninio & Pinna, 2006).

Interestingly, there has been one fascinating finding about the opposite phenomenon, where elements disappear when presented away from fixation (Ninio & Stevens, 2000). Ninio and Stevens (2000) reported that small elements, such as white disks, when placed at the intersection of a grid become invisible in the periphery. Ninio and Stevens named this the Extinction illusion (see also Araragi & Kitaoka, 2011; McAnany & Levine, 2004). It is possible that Extinction and Honeycomb illusions are due to the same causal factor. However, one important difference is that in the Honeycomb illusion, the pattern is a single texture of connected lines, whereas in the Extinction illusion, the white disks are seen as individual objects on top of a background. Thus, there may be specific aspects of texture extrapolation, or failure thereof, that apply to the Honeycomb illusion but not to the Extinction illusion. We will return to this possibility, and to its implications, in the Discussion section.

## Empirical Observations

We document the illusion using a quasi-experimental procedure. Although observers were naïve and tested individually, they were allowed time to think, comment, and develop their description. This is based on the experimental phenomenology approach (Kubovy, 1999; Kubovy & Gepshtein, 2002). In particular, we strived “to devise experimental conditions such as to make the report as close as possible to how observers would describe their experiences outside of the laboratory, but in a highly controlled environment” (Kubovy & Gepshtein, 2002, p. 18).

We asked a group of 12 observers (8 males, mean age of 35) to look at the illusion on a computer monitor and recorded their descriptions. They all had normal or corrected to normal vision.

### Stimuli and Procedure

The images were generated using python on a Macintosh computer. The side of each regular hexagon was 6 mm. Observers were sitting at approximately 57 cm from the monitor, and each side of the hexagon subtended approximately 0.6° of visual angle. The shapes were defined by thin white lines (282 cd/m^2^) on a medium gray background (19 cd/m^2^). The thin red lines had a luminance of 61 cd/m^2^ and the green lines of 42 cd/m^2^. The texture filled the screen and subtended 36° by 28° of visual angle. Along the horizontal, there were 40 stepped hexagons (20 aligned), and along the vertical, there were 27 hexagons (total 1,080). To provide a fixation mark, the hexagon in the center was filled in white (not shown in Figure 1). Observers were allowed to take as long as they wanted to explore the stimuli, and the description was in their own words. In a few cases instead of saying a number of hexagons, they reported a percentage of the whole screen.

## The Honeycomb Illusion: Static

Observers were asked to describe the texture while maintaining fixation on the central white element. They were told that it was important to describe as fully as possible their experience. They reported that they could see a regular pattern made of many hexagons covering the whole screen. All of the observers reported also seeing lines at the corners of the hexagons, but only near fixation. Nobody said that they could see the lines everywhere, even though they all knew that they were there. Asking for a number of hexagons with lines resulted in a mean value of 8.16. The modal answer (6/12 observers) was that they could only see the lines on the six hexagons around the fixation object.

## The Honeycomb Illusion: Multiple Fixations

Observers were asked to describe whether their experience was similar or different when they fixated an element on the left side of the screen, and then on the right side of the screen. Everybody reported that any location gave the same impression. That is, they could only see the small lines in a region close to fixation. On average, they reported seeing lines on 8.66 hexagons. They were then asked whether by looking at multiple locations they could use their knowledge that lines were present everywhere to “see” these lines away from fixation. Everybody said that they could not make use of this information even after many fixations.

## The Honeycomb Illusion: Hexagons and Lines of Different Color

**Figure 2.**
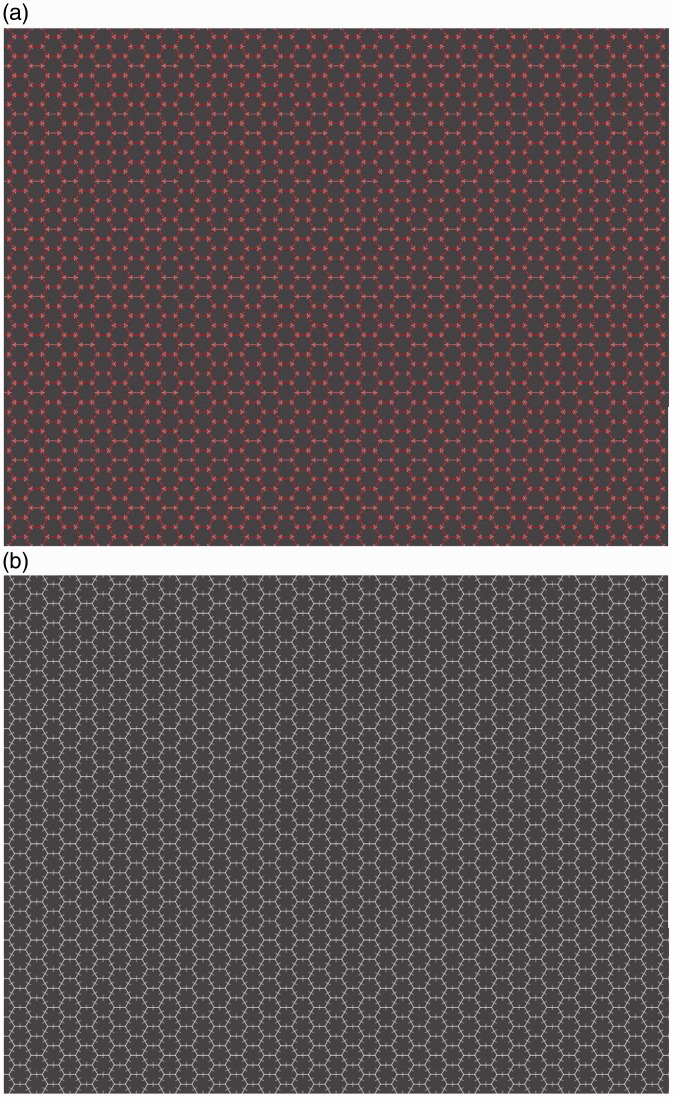
(a) In this example, the color of hexagons and lines differ. The image needs to be seen enlarged and compared with the examples in Figure 1. Here the texture appears uniform. (b) In this example, the lines are in the middle of the sides of the hexagon. Here, the texture appears uniform.

The next image was identical to the first except that while the lines were still white, the hexagons were now red, as shown in Figure 2(a). Every observer said that the color made a difference, and most said that they could see the lines everywhere (9/12 observers). The other three responses were as a proportion or percentage of the screen, and they were 25%, 50%, and 60%. This condition is interesting because it also provides a test of how observers are approaching the task. Suppose that they are compliant with respect to the illusion, they might again report seeing the lines only around fixation to be consistent with the first report. This was not the case. In other observations, we have found that different colors can be used, and a similar effect is produced with a difference in brightness of the lines, that is, when the hexagons are white and the lines are gray. Therefore, it seems important for the illusion that the barbs are seen as attached to or grouped with the hexagons.

## The Honeycomb Illusion: Squares

The next image used squares instead of hexagons. According to all observers, the experience was qualitatively similar to that what they experienced with hexagons. However, the lines were visible in a set of squares that formed some kind of configuration, often described as a cross. On average, they reported seeing the lines on 12.50 squares. The range was between 4 and 30.

## The Honeycomb Illusion: Lines not Placed at the Vertices

In the case of the images used for the previous questions, the lines were placed at the vertices of the hexagons or the squares. To test whether this location is important for the suppression, a new texture was shown in which the lines were orthogonal to the side of the hexagons, as shown in Figure 2(b). Every observer said that the new configuration made a difference compared with the original, and six said that they could see the lines everywhere. The other responses were as a proportion or percentage of the screen, and they were 30%, 50%, 50%, 70%, and 70%. Finally, one observer said that it was too difficult to provide a description. Overall, the visibility of the barbs seems to depend on the location of the lines relative to the corners of the hexagons.

## The Honeycomb Illusion: Local Transients

Local luminance transients are salient and attract attention. It is, therefore, interesting to see what happens when the lines are added to the hexagons. The image without lines was shown to the observers, and they were told that the lines were about to be superimposed. The transition was shown at least twice, or more times when observers asked for it to be shown again. Local transients are present over the whole texture, and they may enhance the possibility of seeing these local features. The descriptions were interesting, as most people claimed to notice some kind of change over the whole screen. A minority (four observers) said that they could see all the lines, but that this percept lasted only a few seconds and soon the lines were fading. After that they were left with the same situation as in the original effect, meaning that they could see only the lines for few hexagons near fixation. The other observers instead said that they never saw the lines over the whole image, instead they reported seeing a global change in brightness, and the lines appeared only near fixation. In terms of the estimate of the size of the region, one person said that she could see 50% of the hexagons with lines (but then they faded to a smaller region). Another said she could see 10 (in the original she had reported seeing 6). Everybody else reported that the region was the same size as in the static case.

## More Demonstrations

In addition to the examples that were shown to the observers, we have provided additional demonstrations on the Open Science Framework project page: https://osf.io/kabyz/. The summary of the images, the Powerpoint files, and the corresponding Figures in the manuscript is in Table 1. We also show examples of the Extinction illusion and Healing Grid. In ShortLongVersions.pptx, it is possible to compare a version of the Honeycomb illusion with shorter and one with longer lines. The region in which the lines are visible increases with the length of the lines. In Translate.pptx, we show that as the texture translate smoothly underneath the fixation spot, the lines appear and disappear just in the location near fixation. In Flicker.pptx, we show a case of transition in which the presence and absence of lines is at 10 Hz. The effect is similar to the single onset used with the observers. In Segmentation.pptx, we show what happens when hexagons and lines are different, specifically in terms of color, luminance polarity, or brightness. When brightness differs the hexagons may become invisible in the periphery.

**Table 1.** Summary of the Images, Powerpoint Files, and the Corresponding Figures.

Image .png	Powerpoint .pptx	Figure in MS	Effect
honeycomb	Flicker Translate	Figure 1(c)	Texture does not appear uniform
honeycombOnlyHex	Flicker	Figure 1(a)	With just hexagons texture appears uniform
honeycombBarbs		Figure 1(b)	With just barbs texture appears uniform
honeycombSquare		–	Similar effect to hexagons
honeycombShorter	ShortLongVersions	–	Shorter barbs decrease the visible region
honeycombLonger	ShortLongVersions	–	Longer barbs increase the visible region
honeycombLongerBarbs	ShortLongVersions	–	With just barbs texture appears uniform
honeycombRed	Segregation	Figure 2(a)	With white barbs and red hexagons the effect is almost gone
honeycombGreen	Segregation	–	With white barbs and green hexagons the effect is almost gone
honeycombPolarity	Segregation	–	With white barbs and black hexagons the effect is almost gone
honeycombBrightness	Segregation	–	With bright barbs and gray hexagons the effect is weak and perhaps reversed, as barbs are now visible but hexagons are only visible at fixation
honeycombMiddle		Figure 2(b)	The effect is absent if lines are not at intersections
healingGrid	Healing	Figure 3(a)	Submitted by Kanai to the Best Illusion of the Year contest 2005, texture appears uniform after adaptation
healingGridReversed	Healing	Figure 3(b)	Reversed version of the Healing Grid
extinctionOriginal	Extinction	Figure 4(a)	From Ninio & Stevens (2000). Disks disappear in the periphery.
extinctionHexagons	Extinction	Figure 4(b)	Extinction illusions with hexagons.
extinctionLarger	Extinction	Figure 4(c)	Extinction illusions with hexagons but disks extend outside lines.
honeycombWithDisks	Extinction	Figure 5	An attempt to make the configuration similar to the Extinction illusion by having very small disks on intersections.
honeycombWithLargeDisks	Extinction	–	The configuration similar to the Extinction illusion but with disks that extend beyond the lines.

*Note.* The first column gives the name of the file (these have the PNG extension). The second column gives names of Powerpoint files using those images (they have the pptx extension). The third column is the Figure in the manuscript, and the last column a brief statement about the effect.

## Discussion

Surprisingly, textures that are uniform can appear nonuniform. This effect occurs in unconstrained, ecologically valid conditions, and as far as we know is present for all observers. The nonuniformity seems due to some form of suppression at the corners of the hexagons. In the examples that we have tested, small lines at corners of simple geometrical shapes, like squares and hexagons, become invisible away from fixation, despite the fact that they are visible when presented on their own. The critical distance from fixation varies with size of the lines.

## Other Related Phenomena

**Figure 3.**
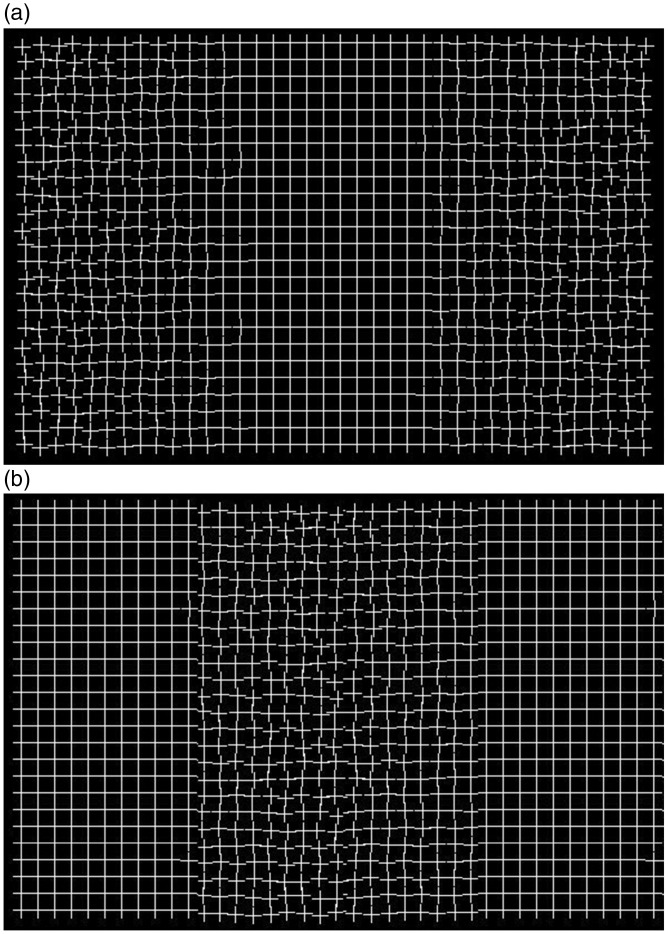
(a) In the Healing grid illusion, the details in the periphery fade and the texture in the periphery end up looking more like the texture at fixation (in the center) illusionoftheyear.com/2005/healing-grid/. (b) The reverse Healing grid (Aggravating grid?) in which irregularity rather than regularity is spreading to the periphery.

Let us compare this phenomenon with two illusions that bear some similarity with it. The first comparison is by contrast with the “Healing Grid” illusion (Figure 3(a)). This illusion was submitted by Kanai to the Best Illusion of the Year contest in 2005 (illusionoftheyear.com/2005/healing-grid/). In the Healing Grid, the regular texture becomes less regular in the periphery. After fixating the center for a few seconds, the regularity spreads into the periphery. The interpretation given by Kanai is that there is a “preference of the visual brain to see regular patterns.” Note that this is a phenomenon in which what is visible at fixation is extrapolated to the periphery, and this extrapolation happens over time as the information in the periphery degrades. We prefer to use the term *extrapolation* rather than *regularization* having examined the reverse situation. In Figure 3(b), the texture is irregular in the center and regular in the periphery. A similar effect takes place after a few seconds of fixation in the center. Although we are not claiming that the effect is perfectly symmetrical, the phenomenon does not seem to be specific to a tendency to maximize regularity, where by regularity we mean unbroken edges and regular spacing.

Perhaps, the most famous example of a uniform pattern perceived differently at fixation is the Hermann Grid (Hermann, 1870) and its variant the Scintillating Hermann Grid (Schrauf et al., 1997). These are strong effects, and they are linked to some form of lateral inhibition, although the full explanation is still debated (Qian, Yamada, Kawabe, & Miura, 2009; Reed et al., 2012). Unlike the Healing Grid, in the Hermann Grid, there is no need for a period of adaptation, and in this respect, it is more similar to the Honeycomb illusion. The main difference is the fact that in the Hermann Grid illusory patches are generated and perceived in the periphery.

## The Extinction Illusion

**Figure 4.**
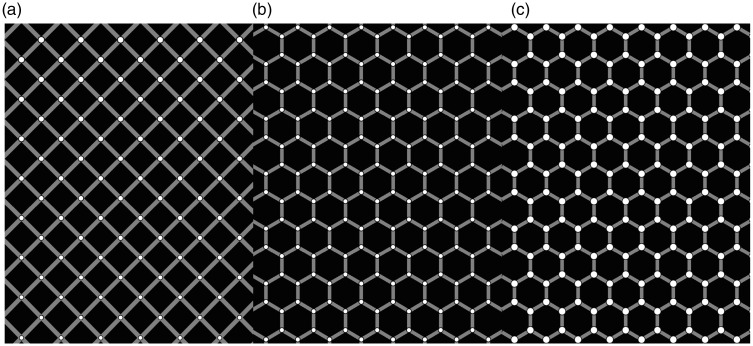
(a) In the Extinction illusion, high-contrast disks are not visible in the periphery (Ninio & Stevens, 2000). (b) A version using hexagons and (c) one in which the disks are too large.

Disks located at the intersection of a grid may become invisible away from fixation. This Extinction illusion, first reported by Ninio and Stevens (2000) as a variant of the Hermann Grid, is remarkably robust and is shown in Figure 4. On the basis of the studies by Ninio and Stevens, we know that the optimal parameters are white disks with a black outline, placed on gray bands (alleys) formed by uniformly spaced black squares. The reverse luminance configuration also works, but it is important that the disks are well defined, that is, have high contrast, and are confined to the gray bands. McAnany and Levine (2004) concluded that there was no role of adaptation, whereas Araragi and Kitaoka (2011) found an increased strength of the effect over time, although the illusion was present even at the shortest of the presentation values tested.

Figure 4 shows a version of the Extinction illusion with hexagons, and one in which the effect is lost as the circles extend beyond the bands. Hexagons are slightly less effective than squares possibly because the disks are at the intersection of three spokes, rather than four. Figure 5 in Ninio and Stevens (2000) shows a powerful version with triangles (thus the disks are at the intersection of six spokes). Conversely, disks not placed at intersections do not disappear.

**Figure 5.**
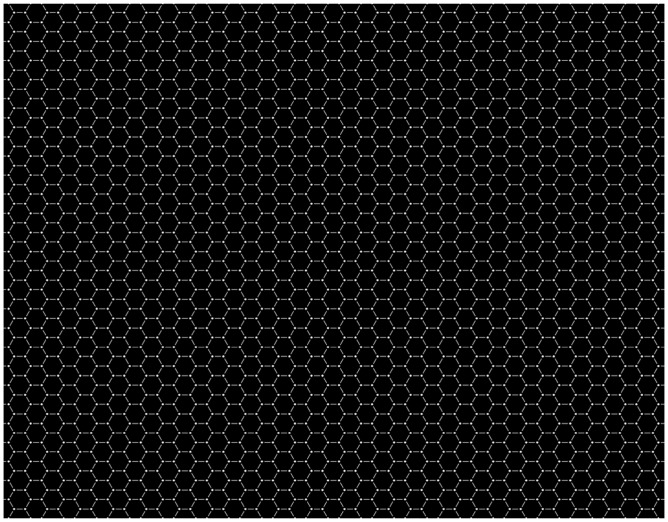
The same image used in Figure 1 is here shown with white disks instead of barbs. This is therefore at the same time the Honeycomb illusion (a percept of a nonuniform texture) and the Extinction illusion (disks disappear in the periphery), but it is not the best example of either.

We were not aware of the Extinction illusion when the Honeycomb illusion was submitted to the Illusion of the Year contest. The phenomena are very similar, for example, in that they do not need adaptation and continue after multiple fixations. The intersection is in both cases a critical location for the disappearance (see Figure 2(b)). Some of the differences are also interesting. There is no simple way to scale up the Extinction pattern to the density used in the Honeycomb illusion. The disks need to be on the bands (lines) and have higher brightness, so this is difficult when the lines are very thin. The best version we could produce is shown in Figure 5. However, note that these tiny disks create segments on the line. In this sense, the pattern is again a uniform texture, unlike the pattern in the Extinction illusion where elements have identities and segregate from the background. Indeed, high contrast is important for this segregation. Barbs in the Honeycomb illusion extend outside the lines, whereas as soon as disks extend outside the bands the Extinction illusion is lost (see Figure 4(c)). Finally, perhaps, the most interesting difference is that in the Extinction illusion lines and disks differ in brightness and are not grouped. By contrast, in the Honeycomb illusion when barbs and hexagons do not group, the effect is much weaker or lost completely as can be seen in Figure 2(a).

## The Role of Memory

It has been known for a long time that people do not combine information from multiple fixations in a fully integrated and detailed representation (e.g., Bridgeman & Mayer, 1983; Irwin, 1991; Irwin, Yantis, & Jonides, 1983). In a famous study, Grimes (1996) asked observers to study and memorize some rich scenes shown using photographs. While the eyes performed a saccade, significant details were changed. Participants, however, failed to detect many of these changes. Subsequent work on the so-called change blindness phenomenon has demonstrated that large changes in a scene are also missed if disruption is introduced by means of global or local luminance transients (e.g., O’Regan, Rensink, & Clark, 1999; Pashler, 1988; Rensink, O’Regan, & Clark, 1997).

In an interesting review of possible explanations for change blindness, Simons (2000) distinguished five processes: (a) overwriting due to masking, (b) first impressions, in the sense of a failure to update, (c) no representation, (d) no comparison, and (e) feature combination. The idea of no representation is particularly interesting, and it has a long history with contributions from artificial intelligence (Brooks, 1991) and philosophy (Dennett, 1991). Fundamentally, it suggests that the world can serve as storage and relevant information is gathered only if and when necessary (see Ballard, Hayhoe, Pook, & Rao, 1997).

The Honeycomb illusion shows a loss of details in patterns away from fixation. Given the well-understood variation in spatial resolution of the retina from the center to the periphery, it could be argued that this effect is not surprising, but it is interesting for at least two reasons. First, the texture is highly regular and without discontinuities. This regularity could be used by the system to interpret the scene as retaining in the periphery features that are visible at fixation. This does not happen. We have contrasted this effect with the Healing Grid, but we can also consider a parallel with color vision. When observers look at a red surface extending to the whole visual field, color information is not available in the periphery which feed mostly into the color-blind magnocellular stream (i.e., Abramov, Gordon, & Chan, 1991), but in the percept, there is a sense that the same color extends continuously away from fixation. With the honeycomb pattern instead, a different mechanism comes into play that prevents this extrapolation. This is taking place despite the fact that regular textures are common in terms of natural statistics, and the presence of fine details only in a small region of an otherwise uniform surface is rare. Therefore, the percept appears to go against any statistical knowledge that the system may have.

If the system does not extrapolate from central vision maybe it can rely on information gathered over time from previous fixations. The hypothesis is that memory plays a critical role in our representation of an extended scene. That is, the world itself serves as a memory store (Brooks, 1991; Dennett, 1991; O’Regan, 1992; Stroud, 1955). However, this role of memory fails to materialize in the Honeycomb illusion. Any of several fixations confirm that the fine details are present everywhere in the scene, and yet the fine details immediately disappear in the periphery with a new fixation.

## Peripheral Information and Crowding

The inability to discriminate a shape in the context of other shapes nearby has been studied extensively. Here, we would like to explore the relationship between the Honeycomb illusion and the other literature. An important phenomenon is known as crowding: the inability to recognize objects in clutter (Levi, 2008; Parkes, Lund, Angelucci, Solomon & Morgan, 2001; Pelli & Tillman, 2008). Thus, both the Extinction and Honeycomb illusion may be related to crowding, in particular since the Honeycomb illusion may involve a regularization process that promotes consistent appearance among adjacent objects in the periphery. Specifically, all elements in the periphery appear as regular hexagons. In crowding, the most effective spacing varies with eccentricity, thus predicting that if spacing is constant as in the Honeycomb illusion the effectiveness of crowding should quickly increase. This may explain the smooth transition from a region within which the lines are visible to the region where they disappear.

However, some fundamental differences should be noted. In crowding, features of elements are jumbled, and strong spatial distortions are observed. However, the visibility of the features itself is not comprised—contrary to the Honeycomb illusion, where the barbs in the periphery are invisible whereas the hexagons are clearly visible and appear, if at all, only slightly distorted. This is true for all explanations of crowding. For example, in feature pooling and compulsory averaging models (e.g., Parkes et al., 2001; Pelli & Tillman, 2008), the barbs would be visible but may be perceived at locations different to where they were presented. The same is true for grouping (Herzog, Sayim, Chicherov, & Manassi, 2015) and substitution models (Strasburger, Harvey, & Rentschler, 1991). Discrimination of the spatial positions of the barbs or identification of barb features may strongly be hampered but the barbs themselves should be visible. The case of red hexagons and white barbs, however, does suggest a role of grouping. In that case, hexagons and lines separate (i.e., hexagons and lines are not grouped), and the Honeycomb illusion disappears.

Another difference is the radial–tangential anisotropy of crowding. The crowding effect is much stronger for radially positioned flankers (i.e., along spokes originating at fixation) than tangentially positioned ones. So far we have not observed any anisotropy for the Honeycomb illusion, although this needs further direct testing.

It has been shown that crowding is not the same as lateral inhibition and surround suppression (Petrov, Popple, & McKee, 2007), and in the second case, stimuli are indeed rendered invisible by contextual elements. However, there are similarities between the two phenomena, including the radial–tangential anisotropy and the fact that size does not affect crowding (Tripathy & Cavanagh, 2002) or surround suppression (Petrov & McKee, 2006). Studies of masking and surround suppression use mainly low contrast targets whereas the contrast of the barbs was high. Moreover, why should only the barbs be suppressed and not the hexagons? And as a last point, lateral inhibition and surround suppression should be scale invariant, that is, mutual inhibition in the fovea should be similar to the periphery, even though peripheral resolution is worse than the foveal one.

To be clear, we do not doubt that the Honeycomb illusion is the result of some kind of suppression. The same is probably true for the Extinction illusion. We are only pointing out some specific aspects of the phenomenon. The focus of our report is not as a detailed study of the process, but a discussion of an important implication of the suppression that takes place for these stimuli: the lack of texture uniformity in the percept.

## Conclusions

Regular grids provide many interesting perceptual phenomena, including illusory patches and extinction effects, that is, the disappearance of some high contrast objects. Here, we have introduced and discussed a grid phenomenon that we call the Honeycomb illusion. We have focussed on the fact that the stimulus is a uniform texture, which is not perceived as uniform. This lack of uniformity is a problem for (a) any hypothetical extrapolation mechanism (from central vision to the periphery), for (b) an inferential mechanism based on prior expectations and natural statistics, as textures tend to be uniform, and also (c) a failure of any memory-based scene representation constructed from successive fixations.

In the case of the Hermann grid, extra features (spots) are visible in the periphery, while in the Honeycomb illusion, some features disappear in the periphery. Similarly, in the Extinction illusion features disappear in the periphery, although these features are separate objects on top of a texture. In all these cases, what we see at fixation is accurate, but moving the eyes to multiple locations and thus “discovering” that the image is uniform does not help in perceiving a uniform image. Thus, we suggest that the Honeycomb illusion constitutes a useful testbed for studying how textures are perceived. Some global features such as color and closed objects (e.g., hexagons) are perceived as unchanged from central to peripheral locations. Other features, such as local details (orthogonal lines) can instead be masked or suppressed, leading to the percept of a nonuniform texture.
